# Serum Myeloperoxidase Activity, Total Antioxidant Capacity and Nitric Oxide Levels in Patients with Chronic Otitis Media

**DOI:** 10.1007/s00232-013-9561-8

**Published:** 2013-06-12

**Authors:** Mehmet Fatih Garça, Mehmet Aslan, Bakır Tuna, Ahmet Kozan, Hakan Cankaya

**Affiliations:** 1 Department of Otorhinolaryngology, Yuzuncu Yıl University, Van, Turkey; 2 Department of Internal Medicine, Yuzuncu Yıl University, Van, Turkey; 3 Department of Clinical Biochemistry, Gazi State Hospital, Samsun, Turkey

**Keywords:** Chronic otitis media, Cholesteatoma, Myeloperoxidase activity, 4-Hydroxynonenal, Malondialdehyde, Total antioxidant capacity, Nitric oxide

## Abstract

It has been suggested that oxidative stress may play an important role in the pathogenesis of chronic otitis media (COM), but the role of oxidative stress in the pathogenesis of COM has not yet been fully explored. Therefore, the aim of this study was to investigate serum myeloperoxidase (MPO) activity, 4-hydroxynonenal (4-HNE), malondialdehyde (MDA), total antioxidant capacity (TAC) and nitric oxide (NO) in patients with COM. Sixty-one patients with COM and 30 controls were enrolled in the present study. Patients were divided into two groups according to the presence (*n* = 21) or absence (*n* = 40) of cholesteatoma. Serum MPO activity and 4-HNE, MDA and NO levels were significantly higher in patients with COM than controls (for all, *p* < 0.001), while TAC levels were significantly lower (for all, *p* < 0.001). Serum MPO activity and MDA, 4-HNE and NO levels were significantly higher in patients with cholesteatoma than in those without cholesteatoma, while TAC levels were significantly lower; but the difference between groups was not statistically significant (*p* > 0.05). Increased oxidative stress seems to be associated with decreased antioxidant levels in patients with COM. Thus, increased oxidative stress may play a role in the pathogenesis of COM. It is believed that the administration of antioxidant vitamins such as A, C and E may be useful in preventing and treating COM.

## Introduction

Chronic otitis media (COM) is the inflammation of the mucosal linings of the airy spaces of the middle ear and the temporal bone, accompanied by perforation of the tympanic membrane with a duration of more than 3 months. The form characterized by accumulation of keratinizing squamous epithelium in these spaces is called COM with cholesteatoma (CCOM) (Semaan and Megerian 2006).

Although many factors have been held responsible for the tissue damage caused by this chronic process, the pathophysiology is still not clear. Many factors implicated in the pathophysiology of COM, such as osteoclastic activity, cytokines, chronic inflammation, endotoxins and products of lipid peroxidation, also cause the production of free oxygen radicals (Semaan and Megerian 2006; Ursick and Fayad 2012). According to the current data, oxidative stress plays a role in the pathophysiology of various conditions such as cancer, cardiovascular diseases, rheumatoid arthritis, ischemia/reperfusion, chronic adenotonsillitis and aging (Koc et al. 2011; Sezer et al. 2012). Investigations have demonstrated that oxidative stress is active in the pathophysiology of acute otitis media, otitis media with effusion and tympanosclerosis (Karlidag et al. 2004; Yilmaz et al. 2004a, b).

The human organism has a complex defense system that protects against the activity of reactive oxygen species (ROS); this is referred to as the “antioxidative system.” Oxidative stress constitutes the basis for many diseases and may account for the severity of systemic and oral disease complications (Vertuoni et al. 2004). It has been indicated that decreased antioxidant system activities and increased ROS production may play a role in the pathogenesis of ROS (Gaté et al. 1999). It has been demonstrated that ROS can directly cause oxidative injury to cells by damaging nucleic acids, proteins, lipids and cell membranes in tissues (Yilmaz et al. 2004a, b).

Lipid peroxidation is the reaction in which the membrane proteins and receptors, as well as their bound enzymes, are inactivated through disintegration of cell membrane integrity (Young and Parthasarathy 1994). In this reaction, polyunsaturated fatty acids are hydrolyzed into biologically active aldehydes and carbonyl compounds. Among these substances, the most important are malondialdehyde (MDA) (Aktan et al. 2003) and 4-hydroxynonenal (4-HNE) (Uchida 2003).

Myeloperoxidase (MPO) is an oxidative enzyme with antimicrobial activity, which uses H_2_O_2_ to produce hypochloric acid and other toxic substances in neutrophil phagolysosomes. It has been reported that MPO plays important roles in chronic processes such as neurodegenerative diseases and atherosclerosis (Podrez et al. 2000; Vita et al. 2004). Nitric oxide (NO), which has both antioxidative and pro-oxidative properties, particularly increases in inflammatory diseases (Joshi et al. 1999; Yilmaz et al. 2004a, b). It also plays an important role in the chronic stage of various diseases (Koc et al. 2011; Sezer et al. 2012).

Although the relationship between oxidative stress and COM has been investigated in several studies (Min et al. 1999; Westerveld et al. 1997), the role of oxidative stress in the pathogenesis of COM has not yet been fully explored. Therefore, the aim of this study was to investigate serum MPO activity and 4-HNE, MDA, total antioxidant capacity (TAC) and NO in patients with cholesteatomatous and noncholesteatomatous COM.

## Methods

### Subjects

This prospective study was conducted in the Department of Otorhinolaryngology at Yuzuncu Yil University (Van, Turkey) between October and December 2011. The study was approved by the Medical Ethics Committee, Faculty of Medicine, Yuzuncu Yil Universty; and informed consent was obtained from the patients. In this study, 61 patients with COM (35 female, 26 male) and 30 healthy controls (15 female, 15 male) were enrolled. Patients were divided into two groups according to the presence of cholesteatoma. Twenty-one patients had cholesteatoma and 40 did not.

None of the patients were receiving regular antioxidant vitamin supplements, such as vitamins E and C. We excluded patients who had acute infections or systemic disease. In addition, patients were not receiving any drugs and were not smoking or consuming alcohol.

The control group consisted of 30 healthy subjects who were asymptomatic with an unremarkable medical history and normal physical examination. None of the control subjects were receiving antioxidant vitamin supplementation, such as vitamins E and C. In addition, subjects were not receiving any drugs and were not smoking or consuming alcohol.

The study protocol was carried out in accordance with the Helsinki Declaration as revised in 2000. The study protocol was approved by the local ethics committee.

### Blood Collection

Following a 12-h fasting period, blood samples were collected at 8:00 and 11:00 a.m. after an overnight fasting period. Blood samples were collected into empty tubes and immediately stored on ice at 4 °C. The serum was then separated from the cells by centrifugation at 3,000 rpm for 10 min. Serum samples were stored at −20 °C until they were used for measurement of MPO activity and TAC, MDA, NO and 4-HNE levels.

### Measurement of Serum MPO Activity

MPO activity was measured by the method of Kruidenier et al. (2003). The sample was homogenized and incubated in 0.5 % hexadecyl trimethylammonium bromide (pH 5.5) and 0.026 % ortho-dianisidinedihydrochloride plus 0.018 % H_2_O_2_. The reaction period was 30 min. The reaction was specifically confirmed by sodium azide (0.1 mM). All analyses were performed and controlled twice. Values were recorded as units per milliliter per milligram. MPO activity was expressed as units per liter of serum.

### Measurement of Serum Lipid Peroxidation Levels

MDA was analyzed by a spectrophotometric method. The sample (0.2 ml) was mixed with 0.8 ml of tamponated phosphate and 25 μl of butylated hydroxytoluene (pH 7.4). Then, 0.5 ml of 30 % trichloroacetic hydrobarbituric acid was added to the prepared solution, which was kept on ice for 2 h and then centrifuged at 2,000×*g* for 15 min at 25 °C. After centrifugation, ethylene diamine tetraacetic thiobarbituric acid (EDTA, 0.075 mm) and 1 % thiobarbituric acid (0.25 ml of 0.1 mol/l) was prepared for every milliliter. The supernatant was kept in boiled water for 15 min and left to cool at room temperature. Spectrophotometric measurement was performed on the most recently prepared supernatant at a 532 nm wavelength. The results were expressed as nanomoles per milliliter.

### Measurement of TAC

Serum TAC levels were measured spectrophotometrically (Genesys 10 UV Scanning UV/VIS Spectrophotometer; Shimadzu, Kyoto, Japan) at 660 nm using kits (Rel Assay Diagnostics kit; Mega Tıp, Gaziantep, Turkey) developed by Erel (2004). This method was based on the bleaching of the distinct color of the 2,2′-azino-bis(3-ethylbenzothiazoline-6-sulfonic acid) radical cation via the action of antioxidants. The results were expressed as millimoles of 6-hydroxy-2,5,7,8-tetramethylchroman-2-carboxylic acid equivalent per liter.

### Measurement of Serum NO

Sample NO levels were measured by Griess reagent according to the method of Moshage (2009). In the first stage a nitrate reductase reaction was performed in order to convert nitrate in the sample. Components from nitrogen that were purple in color were developed by Griess reagent in the second step. Zinc sulfate was added in this formation and centrifuged at 10,000 rotation for 5 min. Measurements were performed using a chromatographic spectrometer at 450 nm wavelength. The measured values were recorded as millimoles per liter per milligram.

### Measurement of Serum 4-HNE

4-HNE was measured with an enzyme-linked immunosorbent assay. Levels were recorded as nanomoles per liter per milligram of protein.

### Statistical Analysis

The results were expressed as mean ± standard deviation. Nonparametric continuous variables were compared with the Mann–Whitney *U* test. Parametric variables were compared using Student’s *t* test. The *χ* ^2^ test was used to compare gender in COM subjects and controls. Pearson’s correlation analysis was used to analyze the extent of the association between serum MPO activity and levels of 4-HNE, MDA, TAC and NO. The results were considered statistically significant at *p* < 0.05. The data were analyzed using SPSS for Windows (version 11.0; SPSS, Inc., Chicago, IL).

## Results

There were no statistically significant differences between COM subjects and controls with respect to age and gender (*p* > 0.05) (Table 1).

**Table 1:** Demographic characteristics of the two groups in this study

Variable	Patients	Control
	(*n* = 61)	(*n* = 30)
Age (years)	22.4 ± 12.2	25.3 ± 4.99
Gender (male/female)	26/35	15/15

Values are mean ± SD

Serum MPO activity and MDA, 4-HNE, and NO levels were significantly higher in patients with COM than controls (for all, *p* < 0.001), while TAC levels were significantly lower (*p* < 0.001) (Table 2).

**Table 2:** Oxidant and antioxidant values for the two groups in this study

Parameters	Patients	Control	*p*
	(*n* = 61)	(*n* = 30)	
MDA (nmol/ml)	4.85 ± 1.58	1.72 ± 0.40	0.001
NO (mmol/l/mg)	11.7 ± 2.60	4.07 ± 2.22	0.001
MPO (U/l)	25.2 ± 6.03	10.6 ± 1.96	0.001
4-HNE (nmol/l/mg)	17.1 ± 3.39	6.27 ± 2.16	0.001
TAC (mmolEq/l)	97.0 ± 13.7	433 ± 130	0.001

Values are mean ± SD

*MDA* malondialdehyde, *NO* nitric oxide, *MPO* myeloperoxidase, 4-*HNE* 4-hydroxynonenal, *TAC* total antioxidant capacity

Serum MPO activity and MDA, 4-HNE, and NO levels were significantly higher in patients with cholesteatoma than in those without, but the difference between groups was not statistically significant (for all, *p* > 0.05) (Table 3).

**Table 3:** Oxidant and antioxidant values in patients with and without cholesteatoma

Parameters	Chronic otitis media without cholesteatoma (*n* = 40)	Chronic otitis media with cholesteatoma (*n* = 21)	*p*
MDA (nmol/ml)	4.58 ± 1.21	5.35 ± 2.06	0.123
NO (mmol/l/mg)	11.5 ± 2.74	12.1 ± 2.36	0.157
MPO (U/l)	24.5 ± 4.03	26.4 ± 8.65	0.664
4-HNE (nmol/l/mg)	16.9 ± 2.77	17.6 ± 4.39	0.537
TAC (mmolEq/l)	96.0 ± 12.5	98.8 ± 15.9	0.658

Values are mean ± SD

*MDA* malondialdehyde, *NO* nitric oxide, *MPO* myeloperoxidase, 4-*HNE* 4-hydroxynonenal, *TAC* total antioxidant capacity

## Discussion

To the best of our knowledge, no previous report about the serum MPO activity in COM subjects has been published. Therefore, this study is the first to investigate MPO enzyme activity in subjects with COM.

In the present study, we also assessed TAC, MDA, 4-HNE and NO levels in subjects with COM. In the present study, we found that serum MPO activity was influenced by oxidative stress levels. Thus, increased oxidative stress and increased MPO activity might play an important role in the pathogenesis of COM. We also found that patients with COM had significantly higher serum 4-HNE and NO levels and lower TAC levels than healthy controls. In addition, serum MPO activity, MDA, 4-HNE, and NO levels were significantly higher in patients with cholesteatoma than in those without, while TAC levels were significantly lower; but the difference between groups was not statistically significant.

Lipid hydroperoxides are hydrolyzed into biologically active aldehydes and carbonyl compounds (Uchida 2003). These substances show a correlation with the degree of lipid peroxidation. These substances affect ion transport and alter enzyme activity by affecting the permeability of the cell membrane. MDA can react with the nitrogen bases of DNA to produce mutagenic, genotoxic and carcinogenic effects (Kalender et al. 2004; Placer et al. 1966).

The αβ-unsaturated aldehyde HNE is created by the reaction of reactive oxygen or nitrogen species with arachidonic acid in cellular membranes during inflammation and exposure to pollutants, such as nitrogen dioxide or ozone (Hamilton et al. 1998). 4-HNE is the agent responsible for many pathologies associated with oxidative stress (Uchida 2003).

Under normal physiological conditions, the antioxidant defense systems control the tissue damage caused by oxidants. This defense comprises enzymatic and nonenzymatic systems. The most important antioxidant enzymes are superoxide dismutase (SOD), glutathione peroxidase (GSH-Px) and catalase (CAT); and the most important nonenzymatic antioxidants are glutathione, tocopherol (vitamin E), ascorbic acid (vitamin C), carotene (vitamin A), albumin, bilirubin and uric acid (Aktan et al. 2003; Yilmaz et al. 2004a, b). This whole antioxidant defense in the body is defined as the TAC, which is in balance with oxidants in the healthy organism. In recent years, to determine the oxidative balance, serum TAC, a reliable parameter in the evaluation of antioxidant capacity, has been measured (Baysal et al. 2013).

Min et al. (1999) demonstrated that in chronic sinusitis free radicals caused DNA damage in the cilia of the nasal mucosal lining, leading to a slowing of ciliary activity. Likewise, Westerveld et al. (1997) found significantly low levels of antioxidants in the sinus mucosa in chronic sinusitis.

The DNA damage induced by oxidants in chronic diseases can prevent the synthesis of antioxidant enzymes and glutathione, and consequently, the damage can be worsened by continuation of the process (Kantar et al. 1994; Yilmaz et al. 2004a, b). In chronic infection, antioxidants slowly regress when oxidative stress attains the level of neutralizing antioxidants. Thus, oxidative stress can be both the cause and the result of chronic infections (Westerveld et al. 1997; Zalewski et al. 2000). In their study on patients with chronic tonsillitis, Shukla et al. (2000) demonstrated that prior to tonsillectomy the MDA level was high, whereas SOD and CAT levels were low and that the serum MDA level decreased following tonsillectomy and the antioxidant capacity increased. The authors suggested that oxidative stress could have a role in the pathogenesis of chronic tonsillitis.

During inflammation, the level of free oxygen radicals rises due to the presence of leukocytes at the inflammation site (Parks et al. 1994). With activation of leukocytes, the NADPH oxidase enzyme and the bactericidal MPO enzyme are produced. MPO, an azurophilic granular element of leukocytes, is left in the phagosome following degranulation; and using H_2_O_2_ and chlorine, it produces the oxidant hypochloric acid (HOCl), which has a strong bactericidal effect (Thomas et al. 1995). Thus, MPO, which shows the presence of leukocytes in inflammatory processes, is used as a marker in both acute and chronic inflammatory diseases (Faith et al. 2008). Recent studies have also shown the important role of MPO in nonmicrobial chronic inflammatory processes (Cay et al. 2012; Melikoglu et al. 2012).

In bacterial acute suppurative otitis media, production of the MPO-mediated oxidant HOCl in leukocytes and the dead bacteria can induce an inflammatory reaction that would lead to lipid peroxidation. This process can increase the oxidative stress in the middle ear, damaging the structure of the cilia through damage of cellular DNA and proteins, and can therefore worsen the tissue damage in the eustachian tube and the middle ear. The described process may be a contribution to eustachian tube dysfunction, the most blamed factor in the pathophysiology of COM. Furthermore, occlusion of the eustachian tube, with the pressure increase in the middle ear, can cause exudation, venous stasis and local acidosis. During reperfusion, the migrating leukocytes set free the MPO-mediated free radicals for the elimination of necrotic cells. Free oxygen radicals can lead to chronicity of inflammation in the middle ear through peroxidation of phospholipids in the cell membranes of the mucosal linings with consequent damage in lipids, lipoproteins, proteins and DNA (Yilmaz et al. 2004a, b). In our study, the serum MPO activity in patients with COM was determined to be significantly high compared to that of the control group. Moreover, MPO showed high activity in COM due to bacterial infection or physiological causes related to occlusion of the eustachian tube.

In their experimental study, Yılmaz et al. (2004a, b) observed vascular dilation and proliferation, leukocyte infiltration, epithelial thickening, squamous metaplasia, edema and exudation on histopathological examination of the middle ear in otitis media with effusion. This histological picture was related to the exhaustion of antioxidants and overproduction of oxidants and in the long term led to tissue damage in the middle ear and consequently to chronicity. In their experimental study on myringotomized rats, Ozcan et al. (2002) studied the effect of topical *N*-acetylcysteine on the formation of myringosclerosis and measured the serum levels of nitrite/nitrate and the products of MDA and NO formed by lipid peroxidation. The results showed that the rate of sclerotic plaque formation and the levels of MDA and nitrite/nitrate were significantly lower in the group of rats receiving therapy compared with the control group. Takoudes and Haddad (1999) showed the presence of lipid hydroperoxide in middle ear fluid in children with COM and demonstrated the possible role of free oxygen radicals in inflammatory damage in COM in men. Parks et al. (1994) found significantly high levels of lipid hydroperoxide and MDA in the mucosal linings of the middle ear of guinea pigs infected with pneumococci compared with those of normal controls.

An inflammatory response is always essential for the structural and functional restoration of damaged tissues. Inflammation is always present in COM samples. The inflammatory granulation tissue can be seen along the active epithelium (Olszewska et al. 2004). The oxidative status during this process, which is due to lipid peroxidation in damaged tissue, has been demonstrated with the increase in serum oxidants in studies on acute otitis media and otitis media with effusion. During the acute phase of inflammation, with formation of PMN leukocyte–mediated HOCl when there is an increase in oxidative stress induced by endothelial dysfunction, such as that seen in COM, the tissue response can turn to chronicity with subsequent pathological changes.

García Callejo et al. (2000) measured lipid peroxide levels in the effusions of CCOM and COM patients with chronic effusion and in effusion otis media patients; they found lipid peroxide levels to be 10 times higher in ears with chronic effusion. Baysal et al. (2013) evaluated the total oxidant status and levels of the oxidative stress index in COM patients and obtained significantly high values. The same study (Baysal et al. 2013) also demonstrated that the values of the total oxidant status and the oxidative stress index were higher in the cholesteatoma group than in the noncholesteatoma group. It was also reported that the total oxidant status and the oxidative stress index were not the sole causative factors in the etiopathogenesis but that the imbalance between oxidant and antioxidant levels was higher in the cholesteatoma group compared to the noncholesteatoma group (Baysal et al. 2013).

Similar to the results of García Callejo et al. (2000) and Baysal et al. (2013), our study on COM patients showed significantly high values of MDA, 4-HNE and NO, reflecting the oxidative stress by lipid peroxidation in cell membranes in tissues, as well as significantly high values of MPO activity reflecting the oxidative status related to leukocytes at the inflammation site. Furthermore, the TAC levels in patients with COM were significantly low compared to the control group. The levels of oxidative stress were higher in CCOM patients than in COM patients, but the difference was not statistically significant.

In conclusion, we consider that free radicals causing oxidative stress play an active role in the pathogenesis of COM and subsequent tissue damage. For this reason, surgical intervention plus maintenance therapy with antioxidants could be an alternative therapy for patients with COM.
